# Centrifugation-free extraction of circulating nucleic acids using immiscible liquid under vacuum pressure

**DOI:** 10.1038/s41598-018-23766-9

**Published:** 2018-04-03

**Authors:** Hoyoon Lee, Wonhwi Na, Chanhee Park, Kyong Hwa Park, Sehyun Shin

**Affiliations:** 1 School of Mechanical Engineering, Korea University, Seoul, 02841 Republic of Korea; 2 Department of Micro/Nano Systems, Korea University, Seoul, 02841 Republic of Korea; 3 Division of Oncology/Hematology, Department of Internal Medicine, Korea University College of Medicine, Seoul, 02841 Republic of Korea

## Abstract

Extraction of cell-free DNA (cfDNA), which exists at an extremely low concentration in plasma, is a critical process for either targeted-sensing or massive sequencing of DNAs. However, such small amount of DNA cannot be fully obtained without high-speed centrifugation (<20,000 g). Here, we developed a centrifugation-free cfDNA extraction method and system that utilizes an immiscible solvent under single low vacuum pressure throughout the entire process. It has been named Pressure and Immiscibility-Based EXtraction (PIBEX). The amounts of extracted cfDNA by PIBEX were compared with those extracted by the conventional gold standards such as QIAGEN using quantitative PCR (qPCR). The PIBEX system showed equal performance regarding extraction amount and efficiency compared to the existing method. Because the PIBEX eliminates the troublous and repetitive centrifugation processes in DNA extraction, it can be further utilized in microfluidic-sample preparation systems for circulating nucleic acids, which would lead to an integrated sample-to-answer system in liquid biopsies.

## Introduction

Nucleic acid-based molecular diagnosis is a key technology for implementing precision medicine in clinics^1–3^. Especially, cell-free DNA (cfDNA), which freely circulates in the blood stream, has been highlighted as a potential biomarker of liquid biopsy for noninvasive prenatal testing (NIPT)^4–6^ and for cancer management such as diagnosis, prognostication, treatment selection, and monitoring of the disease burden^3,7–9^. The cfDNA assay in clinical fields has become possible with the advance in molecular diagnostic techniques, such as droplet digital polymerase chain reaction (ddPCR)^10–12^, next-generation sequencing (NGS)^13–15^, and genome-wide sequencing^16,17^.

However, cfDNA is a demanding analyte owing to its considerably low concentration in plasma, and thus, it has not been widely adopted for clinical diagnosis and research^9,18^. It was reported that the mean concentration of cfDNA was around 30 ng/mL in a healthy control group^19,20^, whereas it was 180 ng/mL in a cancer patient group^21–24^. Furthermore, circulating tumor DNAs (ctDNAs) account for a fraction as low as 0.01% among the total cfDNA, where its concentration would be below the limit of detection for cancer diagnosis^13,25–28^. Mutations of interest may not be detected owing to sampling noise at low allele fractions of the target genes, because the wild type DNAs shed into the blood stream by normal cells are considerably abundant^13,29^.

Therefore, the efficient extraction of circulating nucleic acids would determine its potential for clinical applications in molecular diagnosis. For instance, the extraction of DNA has been a routine process to measure minor fractions of DNA, such as ctDNA and fetal DNA, in maternal plasma^9,18,30^. However, any loss of the target analytes could result in major sources of experimental error in the precise molecular diagnosis. Unfortunately, even though there has been considerable progress in the molecular diagnostic techniques, sample preparation of cfDNA is not a main issue of molecular diagnostic research and thus, a method for it has not been well developed.

**Figure 1:**
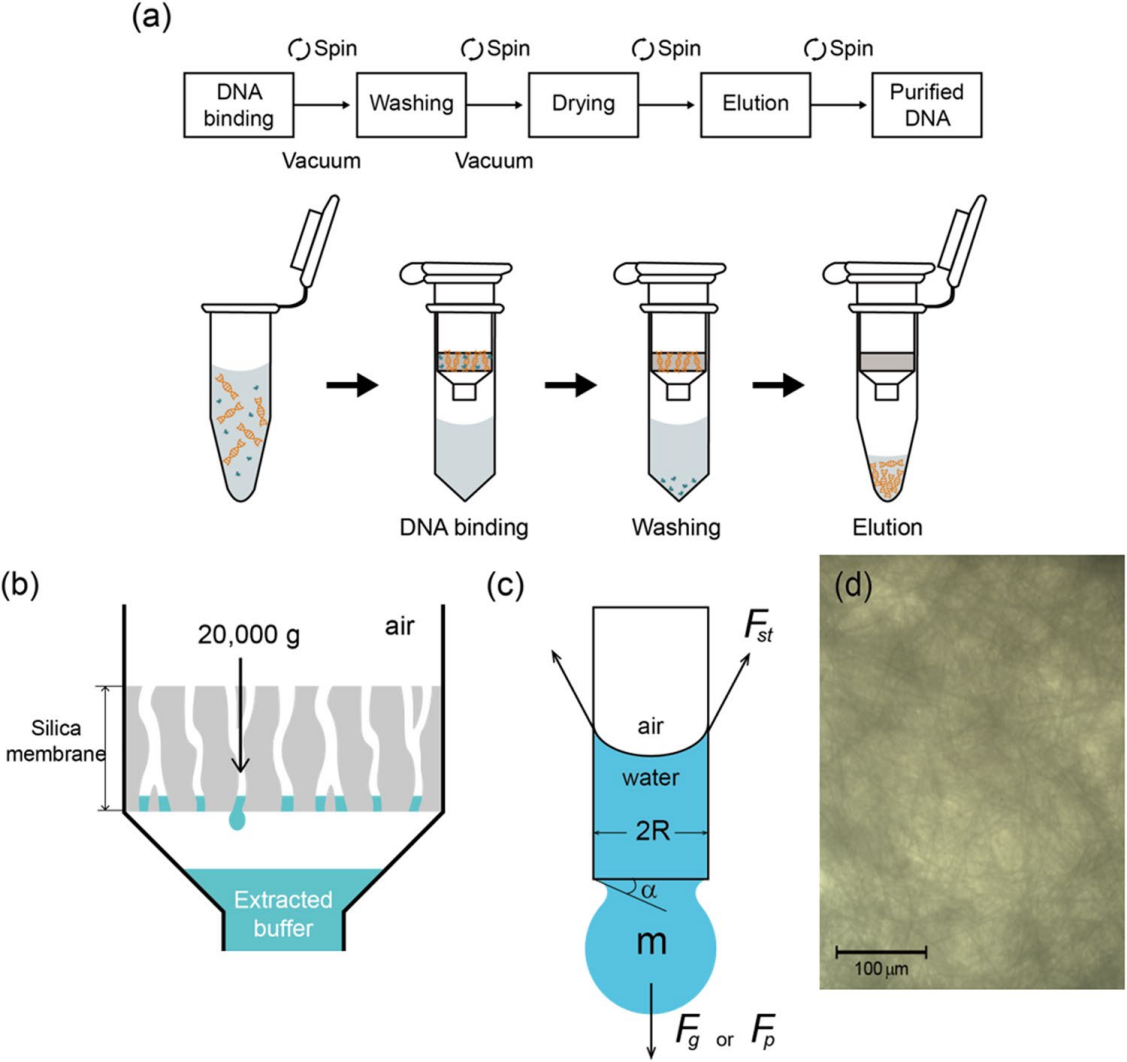
Conventional cfDNA extraction method using centrifugation. (**a**) Workflow of conventional cfDNA extraction using centrifugation. (**b**) Schematic to spin out last residual elution buffer in silica membrane with extremely high g force (20,000 *g*). (**c**) Force balance in a capillary of silica membrane between surface tension force of elution buffer (*F*_*st*_) and gravity force (*F*_*g*_) or pressure force (*F*_*p*_), with capillary radius *R*, angle of droplet neck α, and droplet mass *m*. (**d**) Microscopic picture of silica membrane. The scale bar indicates 100 μm.

Currently available methods of extracting cfDNA are as follows: the affinity-based method using a column or magnetic beads, phenol-chloroform based methods, and filtration based method^9^. In clinical applications, the spin column-based solid phase extraction method is frequently applied to quickly and easily extract nucleic acids^18,30,31^. This method is basically performed with a bench top centrifuge, which requires extremely high centrifugal force to flow various solvents (sample, washing buffer, and elution buffer), as shown Fig. 1. Moreover, the troublous centrifugation process has to be repeated several times for the washing, drying, and eluting processes.

Furthermore, because each step has to be performed manually, involving transferring samples in and out of the centrifuge, the conventional method has several disadvantages, including risk of cross contamination, reduction of work flow consistency, requirement of skilled operators, and high total cost^32^. One applicable way to improve the sensitivity of cfDNA analysis is handling a large sample volume containing more cfDNA^9^, but the loading volume of samples in centrifugation is restricted. Recently, in the spin column-based method, a vacuum-driven extraction of cell free nucleic acids was introduced to control a large sample volume from QIAGEN. However, vacuum cannot substitute the whole process and centrifugation is still required in the drying and elution steps. Given these challenges, there is a clear need for a source technology that can integrate the individual steps, substitute the use of centrifugation, and eventually automate the whole process.

Therefore, we developed a novel DNA extraction method and system that does not require the centrifugation process. This method utilizes a polarity difference between the working liquids for a vacuum-driven flow system. The polarity difference allows the system to operate under a low vacuum pressure throughout the entire process, including the drying and extraction steps. This method was named Pressure and Immiscibility-Based EXtraction (PIBEX). As a proof of principle, the PIBEX was carefully examined with various performance results, which were also compared to those obtained with the conventional gold standard (the QIAamp circulating nucleic acid kit, QIAGEN) using quantitative PCR (qPCR). For the comparative assay, four reference genes obtained from seven individual plasma samples were measured. Because the present study did not intend to develop the membrane required in the process, we used a commercial membrane kit.

## Results

### Analysis of conventional centrifugation-driven extraction

The conventional spin column requires an extremely high *g*-force, as much as 20,000 *g*, especially in the drying and elution steps, as depicted in Fig. 1. We investigated why such a high g-force is required for the drying and elution processes. Most of the liquid can easily flow through the membrane with a low g-force centrifugation, even at 100 g. The problem is the residual water captured in the silica membrane, which is mainly due to surface tension in the microscale membrane pore. For instance, considering a water drop captured in a silica membrane to be a cylindrical capillary with radius *R*, the force balance between surface tension and gravity can be derived. As shown in Fig. 1(c), the surface tension force (*F*_*st*_) in vertical direction is expressed as:

1 $${F}_{st}=2\pi R\gamma \,\sin \,\alpha ,$$

where *γ* and *α* are surface tension (0.072 N/m at 25 °C) and angle of droplet neck, respectively. The gravity force by centrifugation (*F*_*g*_) applied to the droplet is also expressed as:

2 $${F}_{g}=\rho Vcg,$$

where *ρ*, *V*, *c*, and *g* are density, volume of the droplet, a coefficient representing a certain multiple of gravity by rotation speed, and gravitational acceleration: 9.81 *m/s*^2^, respectively. The maximum surface tension force is applied when *α* becomes 90°. Therefore, the force balance between surface tension and gravity is as follows:

3 $$2\pi R\gamma =\rho \frac{2}{3}\pi {R}^{3}cg,$$

and the minimum *R* of the droplet to be detached out from the capillary can be derived as:

4 $$R=\sqrt{\frac{3\gamma }{\rho cg}},$$

Considering the recommended extremely high 20,000 *g* in the spin column manual, the water droplet in a capillary with radius of approximately 30 μm can be flowed out with conventional centrifugation. Interestingly, this simple calculation result was confirmed by examining the pores of the silica membrane, whose sizes range in the tens of micrometers, as observed in Fig. 1(d). Thus, we confirmed that such a high centrifugation, greater than 20,000 *g*, is inevitable for the drying and elution processes.

### Vacuum pressure-driven extraction

**Figure 2:**
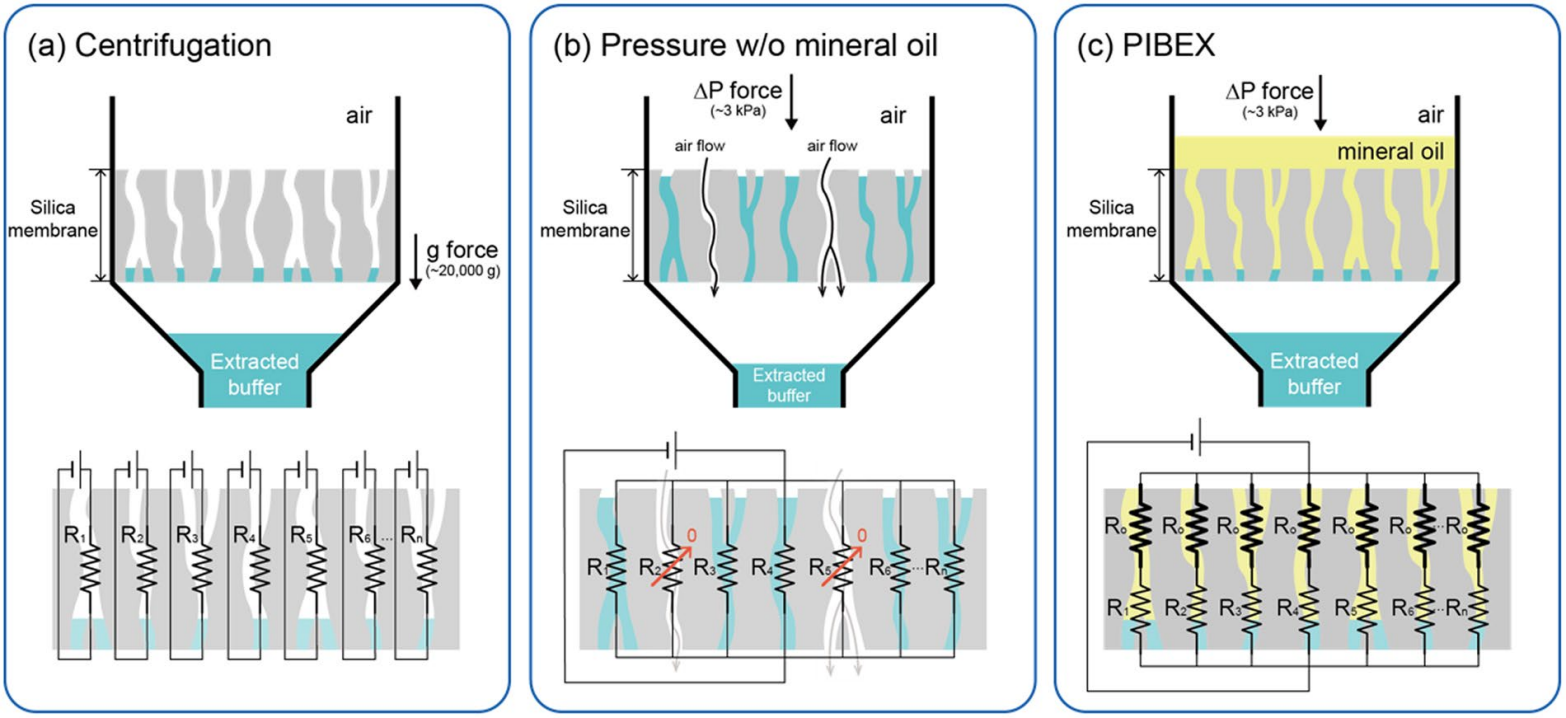
Comparison of extraction mechanisms (**a**) centrifugation, (**b**) pressure without immiscible solvent, and (**c**) pressure with immiscible solvent: PIBEX. The corresponding electrical circuits of each mechanism are depicted. R_n_ and R_o_ are the hydrodynamic resistances of elution buffer and mineral oil through each channel in the silica membrane, respectively (R_o_ ≫ R_n_). R_2_ and R_5_ in (**b**) become negligible owing to air flow. Electrical power corresponds to each driving force.

Meanwhile, the present system adopted a pressure-driven flow system through a silica membrane, as shown in Fig. 2(a). The effective pressure force applied on the cylindrical water droplet captured in the silica membrane is as follows:

5 $${F}_{p}=\pi {R}^{2}{\rm{\Delta }}P.$$

Considering the force balance between surface tension and pressure, the minimum *R* of a capillary to detach droplet under pressure can be derived as:

6 $$R=\frac{2\gamma }{{\rm{\Delta }}P}.$$

Thus, the vacuum pressure to push out the water droplet from a 33-μm radius capillary is theoretically calculated as approximately 4 *k*Pa. Surprisingly, the calculated pressure is somewhat higher than the experimental one (0.7–3.3 kPa). The overestimated value is mainly due to the assumption that highly 3-dimensional structure of membrane pore is approximated as a bundle of capillaries. Anyway, considering a simple generation of the vacuum pressure of 4 kPa, the vacuum pressure method seems to be much easier than ultra-centrifugation method.

After analyzing the required force to push the residual water in a silica membrane, we found that pressure-driven extraction is independent of the mass of a droplet, whereas centrifugation is not. It is well known that surface tension is more dominant with the length scale decreasing to micrometers. To overcome surface tension in a microscale-environment, we need a significant acceleration for a small mass droplet to push out of a membrane capillary, as shown Fig. 1(b) and (c). Thus, the vacuum-driven flow system can be an excellent option as an effective way to completely flow sample and buffers out through the membrane, with significantly low force, even in the drying and elution steps.

### Vacuum-driven extraction using immiscible solvent

Prior to introducing vacuum-driven system, the hydrodynamic flow system through silica membrane can be considered as an electrical circuit. Since the flow resistance through a pore can be considered as an electrical resistance, *R*_*i*_, the system consists of multi-parallel resistances, with the driving potential, as shown Fig. 2. For centrifugation-driven extraction system, the g-force as a driving potential is applied to each pore. It is worthy to note that each flow through a pore is independent of the others and thus it can be regarded as a set of independent serial circuits, as shown in Fig. 2(a). All liquid captured in a silica membrane would be pushed out as long as a sufficiently high g-force as a driving potential is provided. The major resistance is abruptly occurred when pore surfaces begin to contact with air. Then, the surface tension in a microscale pore surface becomes significantly dominant. Thus, the captured liquid in a silica membrane cannot be obtained without supplying such a high g-force due to the surface tension in microscale pore structure.

Meanwhile, even though vacuum-driven systems seem to be suitable for DNA extraction, they have a drawback owing to their own characteristics, as shown in Fig. 2(b). The silica membrane consists of a number of pores holding residual water, and some pores are easily forced to be opened. Owing to the different magnitude of *R*_*i*_ (*i* = 1 to *n*), some pores would be first opened and the flow resistance would become nearly zero, like an electrically shorted line in a parallel circuit. Then, most of air tends to flow though the opened pores, and thus, not all the residual liquid in the silica membrane can be recovered. Thus, a vacuum-driven system cannot push the captured water in the membrane.

**Figure 3:**
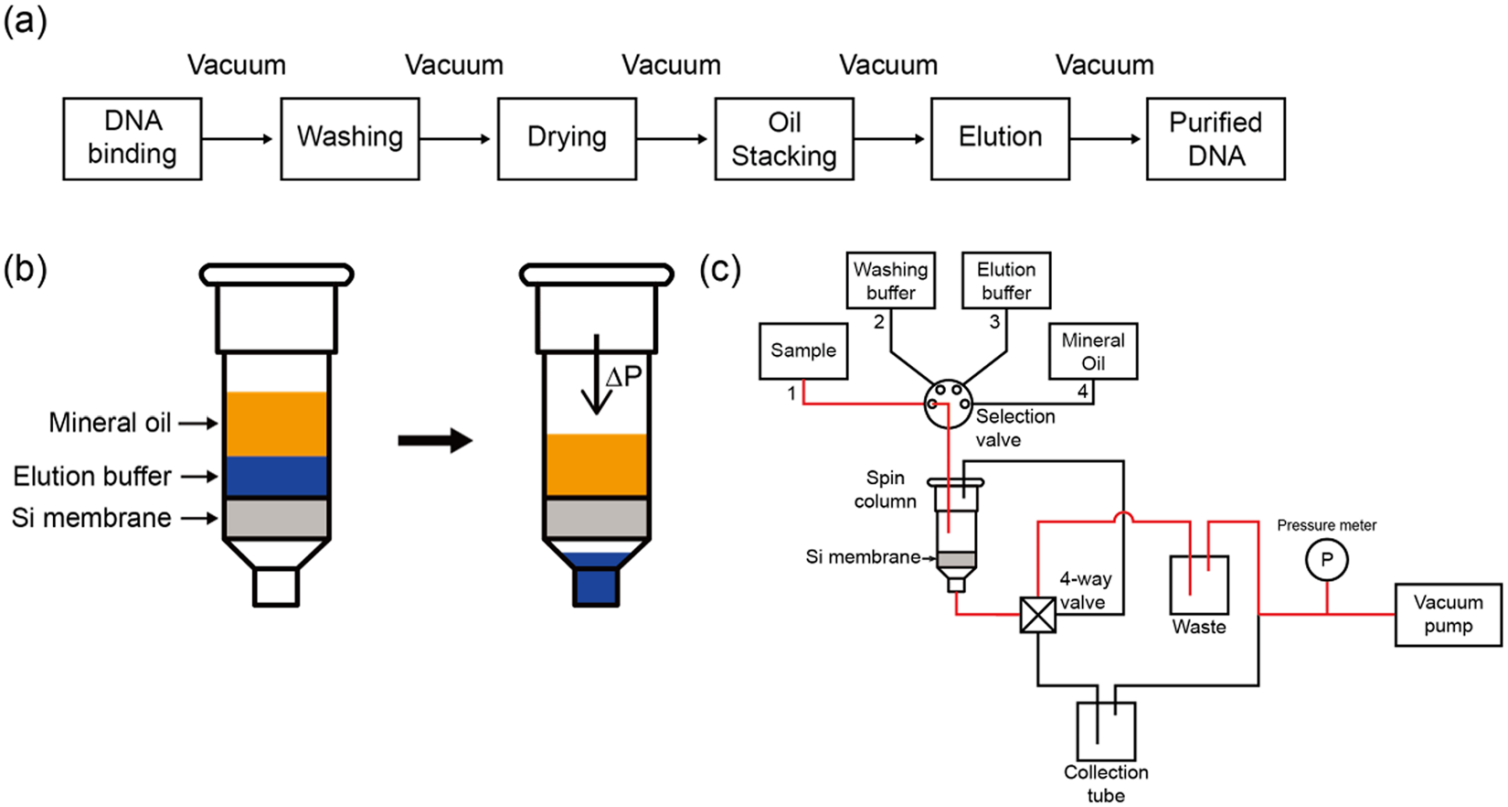
Schematic of PIBEX. (**a**) Workflow for cfDNA extraction by PIBEX. (**b**) Extraction of elution buffer, using immiscible solvent (mineral oil) above the elution buffer layer. (**c**) Schematics of flow channels and valves in PIBEX. The red line represents connected channel.

To solve the problems in the vacuum-driven system, we introduced an immiscible solvent (mineral oil) above the elution buffer layer, as shown in Fig. 2(c). It is worthy to recall that water is a polar liquid and oils are nonpolar ones. The polarity difference between water and oil causes various interesting phenomena. For instance, water and oil do not mix without surfactants. Figure 3 describes the elution mechanism in the PIBEX system. Because the elution buffer is mainly comprised of water, the nonpolar solvent will not be mixed with water. The nonpolar solvent was carefully selected considering post processing, such as NGS or PCR. Mineral oil is a frequently used solvent to generate droplets in a ddPCR assay and to prevent evaporation under high temperature in a PCR assay, whereas it does not interfere in the amplification of target genes. In this research, mineral oil did not affect the qPCR result. Thus, given that mineral oil yields higher viscosity and lower density than water, it could be suitable for stable stacking on the elution buffer. The density and viscosity of mineral oil are 875 kg/m^3^ and 58.6 mPa-s, respectively.

Considering a vacuum-driven system with oil-stacking on the elution buffer, the electric analogy is described in Fig. 2(c). Because mineral oil is about 50 times more viscous than water, the hydrodynamic resistance of mineral oil (*R*_*o*_) becomes much higher than that of water (*R*_*n*_) and thus *R*_*n*_ can be neglected for total resistance (*R*_*o*_ + *R*_*n*_ *~ R*_*o*_). This fact implies that the stacking of mineral oil sets a uniform resistance across all channels. As long as oil exists in the membrane pores, irregular openings will not occur. Thus, with mineral oil of different polarity, all of the residual elution buffer will be completely pushed out, as shown in Fig. 2(c).

### Recovery rate of elution volume

**Figure 4:**
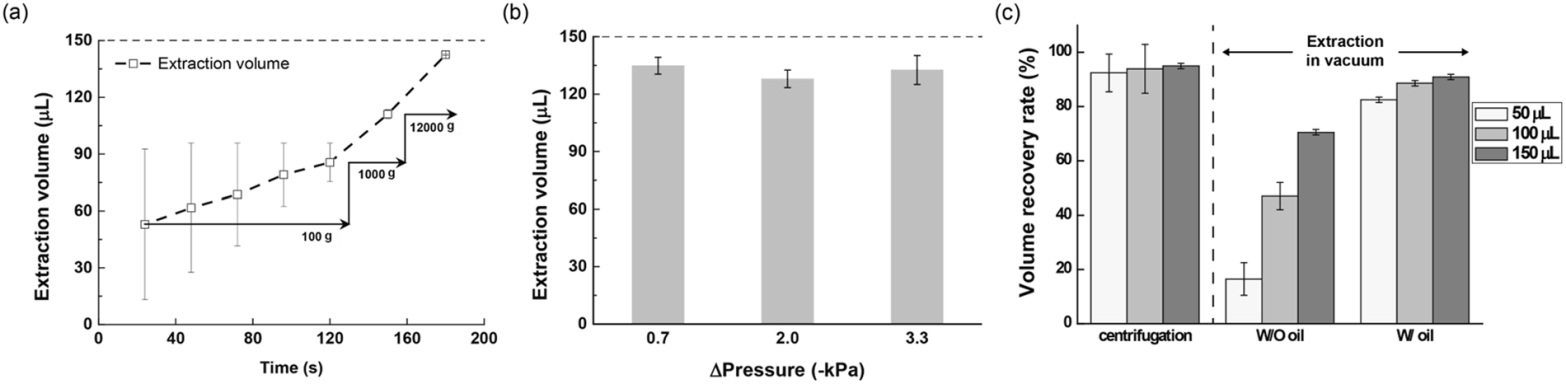
Increase of extraction volume, using vacuum pressure and immiscible solvent. (**a**) Extraction volume of elution buffer in centrifugation with high g force. The error bar is a standard deviation (n = 5). (**b**) Extraction volume of PIBEX with low vacuum pressure in the range of 0.7–3.3 *k*Pa. The error bar represents a standard deviation (n = 3). Dotted lines in (**a**) and (**b**) represent the input volume of elution buffer: 150 μL. (**c**) Volume recovery rate in cases of centrifugation, vacuum driven extraction without oil, and vacuum driven extraction with oil: PIBEX with different input elution buffer volumes, 50, 100, and 150 μm. The error bar represents a standard deviation (n = 3).

The recovery rate of the elution buffer volume was investigated for the present system, PIBEX. A volume of 150 μL elution buffer was loaded in the spin column and extracted using centrifugal force or vacuum pressure force. The g-force was increased stepwise, at 100 *g*, 1,000 *g*, and 12,000 *g*. As a result, centrifugation showed low recovery of elution buffer at a low range of g force, until 1000 *g*, but 90% of the elution buffer was collected at an extremely high g force, 12,000 *g*, as shown in Fig. 4(a). In the present PIBEX system, the extraction volume of elution buffer was high, around 140 μL overall, and the extraction volume was not much affected by the vacuum pressure, in the range of 0.7 kPa to 3.3 kPa, as shown in Fig. 4(b). The recovery rate of extraction volume (%) according to input volume of elution buffer is depicted in Fig. 4(c). Centrifugation showed almost 90% of yield at 12,000 *g*, regardless of input volume. It is because the applied centrifugal force to the water droplet in the silica membrane capillary is dependent only on mass or rotational speed, not on elution volume, as shown in Fig. 2(a) and Equation (2).

The recovery rate of the extraction volume in the vacuum driven system was significantly different depending on the presence of the non-polar solvent, as shown in Fig. 4(c). The recovery rate of oil-free elution was as low as 16% and limited to 70%, even with increased loading volume. This is because air passages are opened while the eluent buffer still flows through the membrane. When the input volume was varied from 50 μL to 150 μL, the amount of un-recovered sample ranged between 30 μL and 50 μL. This means that, on average, the cavity inside the silica membrane can hold about 40 μL of solvent. In addition, although not shown in the data, it has been experimentally proven that the silica membrane of a spin column can absorb 40–50 μL of water.

Surprisingly, the sample captured on the membrane was effectively recovered using a non-polar solvent. Overall, the recovery rate in PIBEX was about 90%, which is almost equivalent to that of centrifugation. The recovery rate of PIBEX did not vary with loading volume. The overall volume yield of the vacuum-driven system with oil was slightly less than that with centrifugation, but was quite effective in recovering the elution buffer, considering the application of a slight vacuum pressure without high-speed centrifugation. Moreover, it was shown that reproducibility of the PIBEX was better than that of centrifugation, as shown in Fig. 4(c).

### Ethanol drying under vacuum pressure

Nucleic acids and silica membrane are both negatively charged and the electrical repulsion interferes with their interaction in the steady state^33,34^. DNA can bind to the silica surface with high concentration of chaotropic salts, such as guanidinium thiocyanate and sodium perchlorate^34,35^. Because residual chaotropic salts may inhibit polymerase amplification for the target gene^36^, chaotropic salts and unwanted impurities should be removed in a washing step. Ethanol is the major component of the washing buffer generally used. However, ethanol also has an inhibitory effect on DNA amplification^36,37^. Therefore, ethanol removal is one of the critical issues in DNA extraction.

In the conventional spin column method, full speed of centrifugation is required to become free of residual ethanol. Ethanol drying using the centrifuge was conducted at 12,000 *g* and it was completed in 50 s, as shown in Supplementary Fig. 1. Similarly, because ethanol can be dried by the air flow driven by vacuum pressure, vacuum-driven drying was also proposed, e.g., with QIAvac 24 plus (QIAGEN, Germany). As a result, room-temperature vacuum drying at 3.3 kPa was completed within 80 s. There was no significant improvement with a 70 °C hot air blower, although temperature of the silica membrane was increased to 43 °C. The drying time increased from 50 s to 80 s but the difference was not critical, and thus, conventional high centrifugal drying could be simply replaced by room-temperature vacuum drying in the present system.

### cfDNA extraction from plasma sample

The amount of cfDNA extracted by the PIBEX system was compared with that by a conventional spin column-based method for cfDNA extraction. The QIAamp circulating nucleic acid kit (QIAGEN) was selected as the gold standard, because its stable and high isolation ability of cfDNA has been verified in recent studies^18,38^. The extraction of cfDNA from plasma samples was quantified by qPCR analysis, measuring the amount of the following genes: TERT, RPPH1, GAPDH, and NAGK.

**Figure 5:**
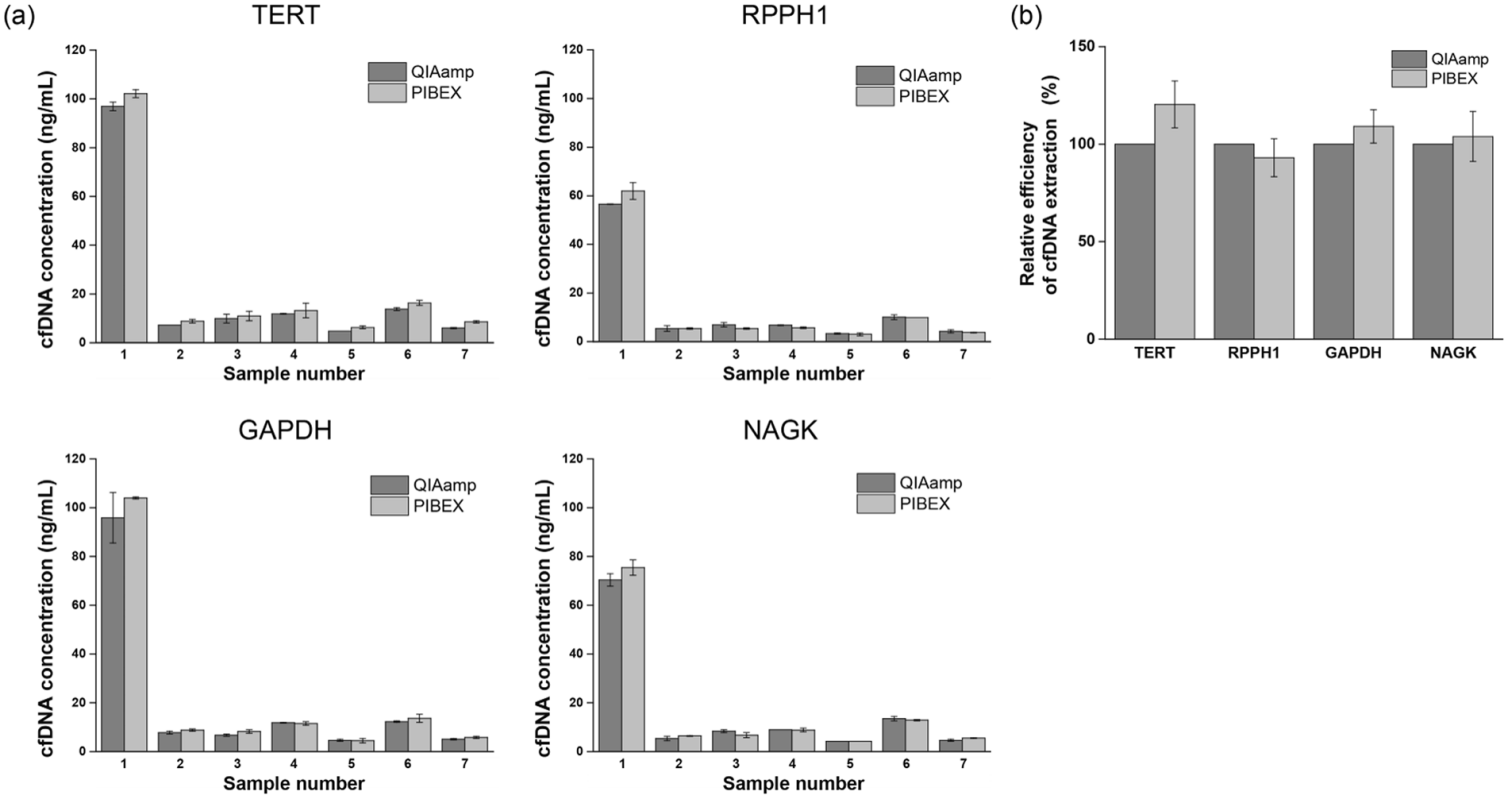
Comparison of extracted cfDNA concentration in qPCR assay. cfDNA concentration in a plasma sample was compared according to extraction methods, QIAamp and PIBEX. cfDNA amount in plasma sample was quantified by qPCR in four reference genes, TERT, RPPH1, GAPDH, and NAGK, in (**a**). The error bar represents standard deviation of triplex qPCR assay in a sample. The relative efficiency of cfDNA extraction in PIBEX was derived based on QIAamp in (**b**). The error bar is standard deviation of samples (n = 7).

The yields of cfDNA, whose unit is ng/mL, are shown in Fig. 5(a). It shows that the selected reference genes could be suitable markers to represent the total cfDNA quantity, because of the similar cfDNA concentration in each sample^18,38^. The extraction amounts of cfDNA in both methods, PIBEX and QIAamp, were in the normal range in the control group, corresponding to a range of 1.8–44 ng/mL^19^, and a low average concentration of cfDNA around 30 ng/mL^19,20^. Furthermore, the characteristic of a considerably high variation of cfDNA concentration in each person^39–41^ was evenly observed in the two methods. The extraction efficiency of cfDNA in terms of percentage is compared in Fig. 5(b). Figure 5(b) shows the relative efficiency of cfDNA extraction of the present system compared to QIAamp, which is the commonly used in laboratories. The results of the reference genes were comparable to the gold standard method. Although there was no statistical significance, cfDNA efficiency was higher in PIBEX than in QIAamp in all genes, except RPPH1. The present system was unable to recover the same amount of extraction as that obtained in the centrifugation in Fig. 4. However, the extraction yield of nucleic acids, which may be more important in precise diagnosis, was equal or even higher. Additionally, we have conducted additional experiments to verify the purity of the extracted DNA for purity of the extracted DNAs. Using a microfluidic electrophoresis equipment (Bioanalyzer-2100, Agilent Genomics), we have analyzed the size of extracted DNAs. As shown in Supplementary Fig. 3(a) and 3(b), QIAamp and PIBEX indicate 172 bp for QIAamp and 169 bp for PIBEX, respectively. Since both results yield nearly identical size of cfDNA peaks, we can claim that the purity of the extracted DNA in PIBEX are equivalent to that of QIAamp. Furthermore, genomic DNA contamination in the large base pair range was not observed in both results.

## Discussion

Surface tension near a solid surface can be considered an adhesive force, attracting water molecules to the solid surface. If the solid surface is wettable, the water molecules are strongly attracted to the surface and its surface would form a meniscus with a sharp angle. These phenomena get significant with decreasing pore size. Furthermore, because the operating principle of the spin column is based on solid phase extraction with a significantly increased surface area, the membrane pore size cannot be simply increased to overcome the surface tension. Thus, a high g-force to overcome the adhesive force of water would be inevitable in such a microscale environment.

The key idea of this study was to introduce mineral oil, which is a nonpolar liquid, to the upper boundary of a polar liquid, the elution buffer (water). They are immiscible with each other owing to the difference in their polarities. In addition, because the density of mineral oil is lower than that of water, it can be stably present on the top surface of water, as shown in Fig. 3(b). By replacing air with oil, the contact angle of water was apparently increased (data are not shown), and thus, the adhesive tension would be significantly decreased. Then, even though a low vacuum pressure is applied, the water droplets caught in the pores of the silica membrane are easily pushed and collected.

In the present study, we developed a vacuum-driven DNA extraction system, which covers the entire process, including the drying and extraction steps. All extraction steps were operated with vacuum pressure without using a centrifuge. It is worthy to recall that we adopted a commercial spin column kit, including reagents, for comparison, excluding the membrane effect. Thus, any other kit can be applied to the present system without restriction. Of course, the extraction performance would be dependent on the quality of solvent composition, pH, reagent volume, materials to use, and method for storage, among other factors.

Moreover, the applicable types of target and samples are only dependent on the product availability. For example, a large spectrum of targets (circulating RNA, miRNA, and viral nucleic acids^42–44^) and samples (human plasma, serum, urine, or other cell-free body fluids^45–48^) can be applicable in our system with a QIAamp circulating nucleic acid kit. Similarly, the extraction efficiency of nucleic acids can be determined by the user kit selection. We emphasize that PIBEX achieved an extraction amount of cfDNA from plasma sample comparable to that achieved by the existing method, as shown in Fig. 5, which refers to the cfDNA extraction efficiency in our system. Furthermore, our system is expected to be easily applicable to genomic or plasmid DNA as well, because they have higher concentration in the sample and larger base pair length than the circulating nucleic acids^9,49^.

Furthermore, our system extracting cfDNA under vacuum pressure bypasses many of the challenges faced by centrifugation, which suffers from cross contamination by manual process, inconsistency of work flow, requirement of an experienced operator, limited input volume, and consequently, high total cost^32^. Although flow generation using vacuum pressure was recently adopted to cover large volume samples, the drying and elution step must be conducted in the centrifuge at extremely high rotating speed, and thus, the work flow of experiments becomes more complicated. However, the conventional centrifugation method, particularly involving drying and elution steps, was efficiently substituted by vacuum pressure with the addition of mineral oil to push out the elution buffer. In the present research, it was theoretically and experimentally verified that the required vacuum force was significantly small.

In general, efficiency of DNA extraction is dependent of surface area of either membrane or beads. The smaller particles are, the larger surface area would be. Additionally, surface functionalization of microbeads would increase the extraction efficiency rather than conventional silica surfaces^50,51^. However, the nanoparticles are rarely used in commercial products and minimum size of microbeads is 1 µm. The rare use of magnetic nanoparticles may be due to the fabrication cost and technical difficulty in collecting nanoparticles with magnetic force. The fabrication of nanoparticles and additional functionalization of bead surfaces would result in increasing cost per unit test compared to membrane methods. In fact, as listed in Table 1, the membrane method yields slightly cheaper price per unit test than magnetic bead methods. However, the membrane method does not selectively extract cfDNA and genomic DNAs and thus additional purification processes using magnetic bead technology are required to obtain pure cfDNA.

**Table 1:** Comparison of cfDNA extraction methods.

Method	Centrifugation method	Magnetic bead methods	Present
Advantages	- Large capacity to handle - Widely used	- Centrifuge not required - Size-selective extraction	- Centrifuge not required - Large capacity to handle - Cross-contamination free
Disadvantages	- Ultracentrifuge required - Purification required	- Laboratory process - Skill required	- Purification required
DNA amount	High	Moderate	High
Cost/test	Moderate	High	N/A

Finally, a single driving force in the DNA extraction method could integrate and automate it in a disposable cartridge or chip. To date, the existing automation systems for nucleic acid extraction have been limited to application of the liquid handler or automated transfer of the spin column into the imbedded centrifuge to substitute a manually labored process^52,53^. This approach is a way to reproduce the conventional centrifugation method as it is, but has limited practical application in clinics and laboratories owing to the large size of the instrument, requirement to purchase an exclusive reagent kit, and high cost of the initial set up and maintenance. If the entire process of nucleic acid extraction is automatically controlled by a united driving force in a disposable cartridge, the total cost could be relatively lower than that with the existing methods. Therefore, this proof of concept study can be further expanded into an eventual sample-to-answer system in liquid biopsies, which could provide a solution for the unmet needs in clinical cancer management and NIPT.

## Methods

### Blood sample preparation (Ethics statement)

The human blood studies were carried out according to the principles of the Declaration of Helsinki. The Institutional Review Board of Korea University approved this study protocol (IRB project number: 2015-12-0016 (ED15318), Seoul, Republic of Korea). All participants provided written informed consent.

To extract cfDNA from a plasma sample, reagents in the QIAamp circulating nucleic acid kit (55114; QIAGEN, Germany) were utilized. The entire process of the centrifugal force-based method using spin column was followed according to the recommended manual from QIAGEN. Blood samples from seven healthy controls were collected in a K2-EDTA tube (Supplementary Table 1). Plasma was isolated at 1,900 *g* for 10 min in a centrifuge (1248; LABOGEN, Denmark) and was re-centrifuged at 12,000 *g* for 15 min; 500 μL of proteinase K was added to 5 mL of plasma in a 50-mL conical tube; 4 mL of lysate-buffer (ACL) containing 1.0 μg of carrier RNA was added. After vortexing for 30 s, the mixture was incubated at 60 °C for 30 min. Then, 9 mL of binding buffer (ACB) was added. After vortexing for 30 s, the final mixture with total volume of 18.5 mL was incubated on ice for 5 min.

### Centrifugal force-based extraction method

Conventional cfDNA extraction was conducted by the processes of DNA binding, washing, drying, and elution, as described in Fig. 1. In this method, the additional extender to handle a large volume of the final mixture (18.5 mL) was inserted into the spin column. The spin column was mounted on a QIAvac 24 plus (QIAGEN, Germany), which was connected to the vacuum pump. The final mixture was carefully applied into the tube extender. In the DNA binding step, the mixture was drawn by vacuum pressure and the tube extender was carefully removed. In the washing step, 600 μL of ACW1, 750 μL of ACW2, and 750 μL of 98% ethanol were sequentially drawn as described above. In the drying step, the spin column was transferred to a centrifuge with a fresh micro tube and the maximum g force was applied at 12,000 g for 3 min. After changing the micro tube to the new one, it was incubated for extra drying in an oven set at 56 °C for 10 min. In the elution, the microtube was replaced by the new one and 150 μL of elution buffer (AVE) was carefully added into the spin column. The spin column was re-transferred to the centrifuge and the maximum *g* force was applied at 12,000 g for 1 min. In the experiment for evaluation of the extraction volume according to the applied *g* forces, 50–150 μL of elution buffer was extracted under a specific *g* force at 100 *g*, 1,000 *g*, or 12,000 *g*.

### Fabrication of PIBEX system

The PIBEX system consists of 6 chambers, a 6-way selection valve, a commercial spin column, and the right angle 4-way valve, collection tube, waste chamber, pressure gauge, and vacuum pump. The sample chamber consists of 30 mL of Luer-Lok syringe (302832; Becton Dickinson, USA) and the other chambers consist of 1 mL of Luer-Lok syringe (309628; Becton Dickinson, USA). The 6-way selection valve (V-241; UPCHURCH, USA) can selectively connect the spin column to a specific chamber. The spin column from the QIAamp circulating nucleic acid kit (55114; QIAGEN, Germany) was mounted in a 1.5-mL microtube reservoir (KRXS-V2; Elveflow, France) and is connected by peek tubing of 1/16-inch diameter (1516 L; UPCHURCH, USA). The bottom of the spin column is connected by swivel barbed to the thread adapter (D-646; UPCHURCH, USA). A check valve (CV-3315; UPCHURCH, USA) connected by tubing connector (P-794; UPCHURCH, USA) was added to prevent backflow. The right angle 4-way valve (V-101L; UPCHURCH, USA) is connected to switch the flow path of the system to the collection tube or waste chamber. The waste chamber of 50 mL Falcon tube (352070; Corning Inc., USA) and the collection tube of 1.5 mL micro tube (MCT-150-C-S; AXYGEN, USA) are separately connected to the 4-way valve. The ends of the waste chamber and collection tube are connected by a Y-barbed connector (P-863; UPCHURCH, USA) with 3/16-inch tubing (LMT-55; SAINT-GOBAIN, France), and it is connected to the vacuum pump (P704AC; GAST, USA), including a pressure gauge to monitor the applied vacuum pressure, with 1/4-inch tubing (KA22-50; INEXUS INC., South Korea).

### Flow channel configuration in PIBEX system

The schematics of PIBEX is shown in Fig. 3. The PIBEX method is based on the processes of DNA binding, washing, drying, oil stacking, and elution, as shown in Fig. 3(a). Figure 3(c) shows DNA binding. A plasma sample flows under vacuum pressure through the selection valve to the spin column, right angle 4-way valve, waste chamber, pressure gauge, and vacuum pump. In the washing phase, the selection valve connects the washing buffer chambers to the spin column. In the drying step, room-temperature air flows through the silica membrane of the spin column under vacuum pressure to dry the membrane completely. Here, the selection valve is connected by the last washing chamber. The elution phase is divided into three steps. For the first step, the elution buffer is loaded on the silica membrane of the spin column. Then, the bottom channel of the spin column is closed and the two top channels of the spin column are connected to the collection tube by switching of the 4-way valve. The spin column acts as a vacuum reservoir where the elution buffer is dropped off by gravity despite air suction, so that the elution buffer could be accumulated on the silica membrane surface. For the second step, the mineral oil chamber is connected to the selection valve and the mineral oil is carefully stacked on the elution buffer layer. At the last step, the bottom channel of spin column is re-connected to the vacuum pump through the collection tube by switching of the 4-way valve. The entire process can be controlled by valves and vacuum, without moving the spin column and using the centrifuge.

### PIBEX method

The final mixture prepared in blood sample preparation was applied in the PIBEX system. 19.5 mL of mixture, 600 μL of ACW1, 750 μL of ACW2, 750 μL of 98% ethanol, 150 μL of elution buffer (AVE) and heavy mineral oil (330760; Sigma-Aldrich, USA) were loaded in each chamber. Each step from DNA binding to washing step was controlled by switching selection valve under 3.3 *k*Pa of vacuum pressure. In the drying step, the selection valve was not switched to connect the last washing chamber and an additional room temperature air flow was applied for 3 min. In the elution step, the right angle 4-way valve was switched to place the elution buffer on the silica membrane, while at the same time the selection valve was switched to the elution buffer chamber. After loading of the elution buffer, the selection valve was switched to the mineral oil chamber. Finally, after stacking mineral oil, the 4-way valve was switched to collect the elution buffer containing cfDNA. When the elution buffer was sufficiently collected in the collection tube, the vacuum pump was turned off, and then, residual vacuum was released by unlocking the mount of the collection tube.

To prevent the cross contamination, silica membrane was replaced with a new one for each test and the other system including chambers and tubes was thoroughly washed with 30 ml of 99% ethanol and dried with air at room temperature for more than 10 min. To confirm cross contamination, the newly obtained sample after washing was analyzed by qPCR and it was confirmed that there is no residual DNA in the previous sample.

### Quantification of cfDNA extraction by qPCR

The amount of total cfDNA extracted by QIAamp and PIBEX method was quantified by qPCR (StepOne™ Real-Time PCR system; Applied Biosystems, USA), using four reference genes; TERT, RPPH1, GAPDH, and NAGK^18^. The standard curve was obtained by qPCR assays in triplicate for each reference gene. Seven points of five-fold dilution series (6.4 pg/μL–100 ng/μL) of human genomic DNA (G3041; Promega, USA) were performed to generate the standard curves. The test of samples from seven healthy controls were performed in triple reactions. 20 μL of reactions containing 2 μL of extracted sample were performed with the Taqman Fast Advanced Master Mix (4444557; Thermo Fisher Scientific, USA) for the entire experiments (Supplementary Table 2). The primer sets and Taqman probes were synthetized by GENOTECH (South Korea). The PCR reaction was performed in two steps; denaturation at 95 °C and annealing at 55 °C. The efficiency of qPCR was derived from the slope of linear regression analysis of cycle threshold (Ct) versus concentration. The concentration of total cfDNA in the plasma was determined by the Ct value of the sample, with comparison of the standard curve.

### Analysis of purity of extracted DNA

The purity of DNA extracted from QIAamp and PIBEX was examined with a microfluidic electrophoresis device (Bioanalyzer 2100, Agilent Technologies, USA). The analysis was performed in a high sensitivity DNA assay chip (5067-4626; Agilent Technologies, USA) with high sensitivity DNA reagents (5067–4627; Agilent Technologies, USA). 5 μL of reaction solutions was initially loaded in the DNA assay chip, then 1 μL of extracted DNA solution was added. The base pairs of the extracted DNA were determined by the time lapse of fluorescence signal compared with the ladders (lower marker; 35 bp and upper marker; 10,380 bp).

Also, we have examined any possible existence of emulsions in the final elution buffer with DLS (dynamic light scattering) device (DLS-8000, Otsuka electronics, Japan). The DLS measurements confirmed that there is no emulsion in the final output biffer.

### Statistics and reproducibility

The statistical analysis performed for quantification of results is indicated in the figure legends. All experiments were independently performed at least three times, unless stated otherwise in the figure legends.

## Electronic supplementary material

Supplementary Information
