# Supplementary material for: Centrifugation-free extraction of circulating nucleic acids using immiscible liquid under vacuum pressure
Source: Sci Rep. 2018 Apr 3;8:5467. doi: 10.1038/s41598-018-23766-9 (PMC5883035; doi:10.1038/s41598-018-23766-9)
Supplement: Supplementary file 1 — Supplementary Information [file 41598_2018_23766_MOESM1_ESM.doc]

**Supplementary information**

Centrifugation-free extraction of circulating nucleic acids using immiscible liquid under vacuum pressure

Hoyoon Lee^1^, Wonhwi Na^2^, Chanhee Park^1^, Kyong Hwa Park^3^, and Sehyun Shin^1^, *

^1^School of Mechanical Engineering, Korea University, Seoul 02841, Republic of Korea

^2^Department of Micro/Nano Systems, Korea University, Seoul 02841, Republic of Korea

^3^Division of Oncology/Hematology, Department of Internal Medicine, Korea University College of Medicine, Seoul 02841, Republic of Korea

*Corresponding author: Sehyun Shin, School of Mechanical Engineering, Korea University, Seoul 02841, Republic of Korea; Tel.: +82 2 3290 3377; Fax: +82 2 928 5825; e-mail: [lexerdshin@korea.ac.kr](mailto:lexerdshin@korea.ac.kr)

**Supplementary Figure 1. Ethanol drying.** Ethanol, which is major component of the washing buffer, was dried out by the following methods. (a) Conventional centrifugation at 12,000 *g*. (b) Vacuum drying with hot air at 3.3 *k*Pa. (c) Vacuum drying with room temperature air at 3.3 *k*Pa. 750 μL of 99.9% ethanol was loaded in a spin column. Mass of residual ethanol in silica membrane of spin column was measured by scale and converted to volume with its density. (d) Comparison of complete drying times. Every experiment was performed in triple. Error bars in this figure represent the standard deviations.


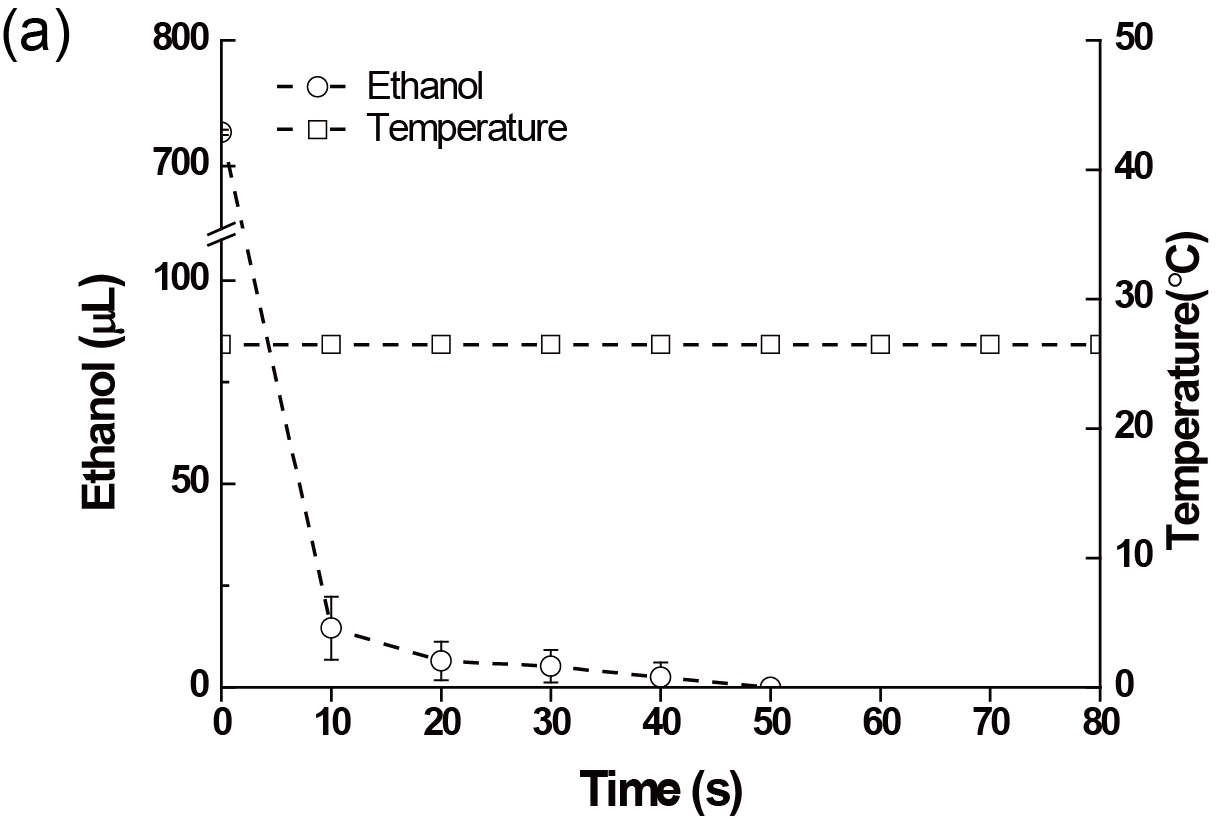

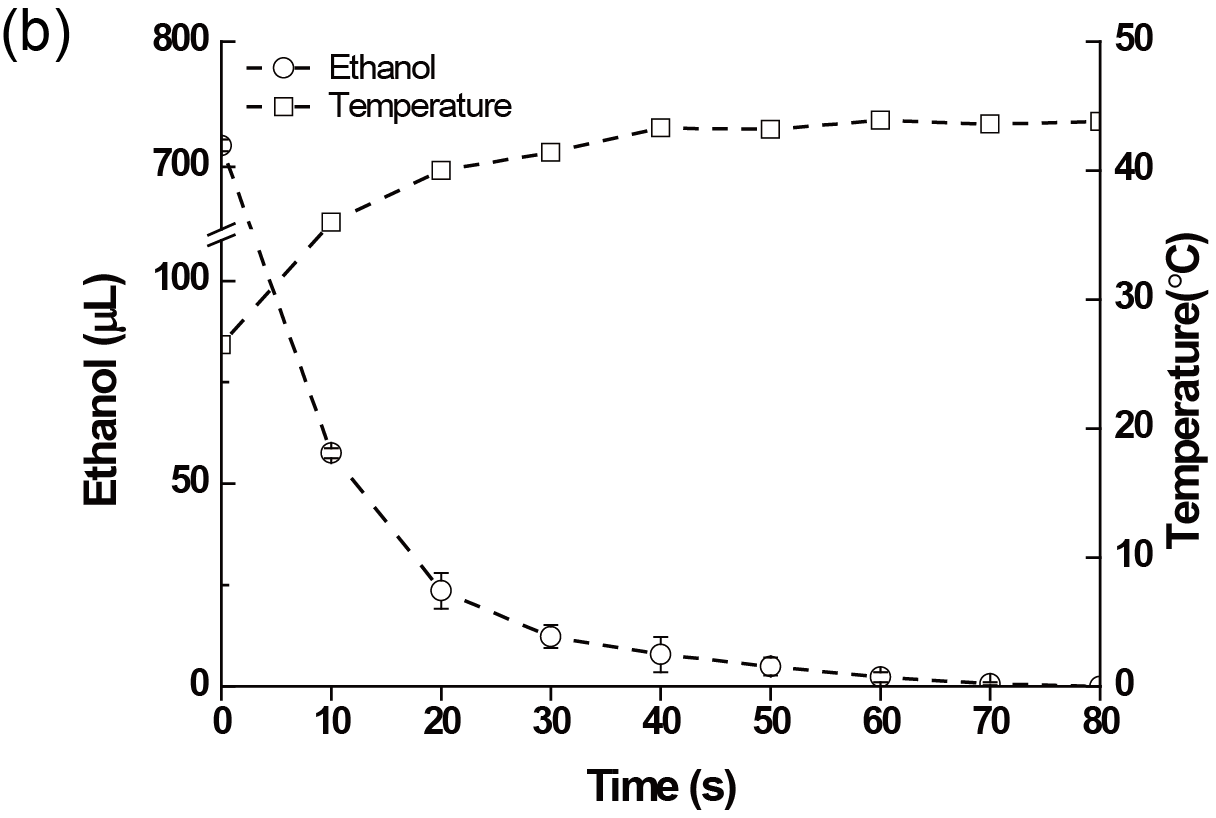


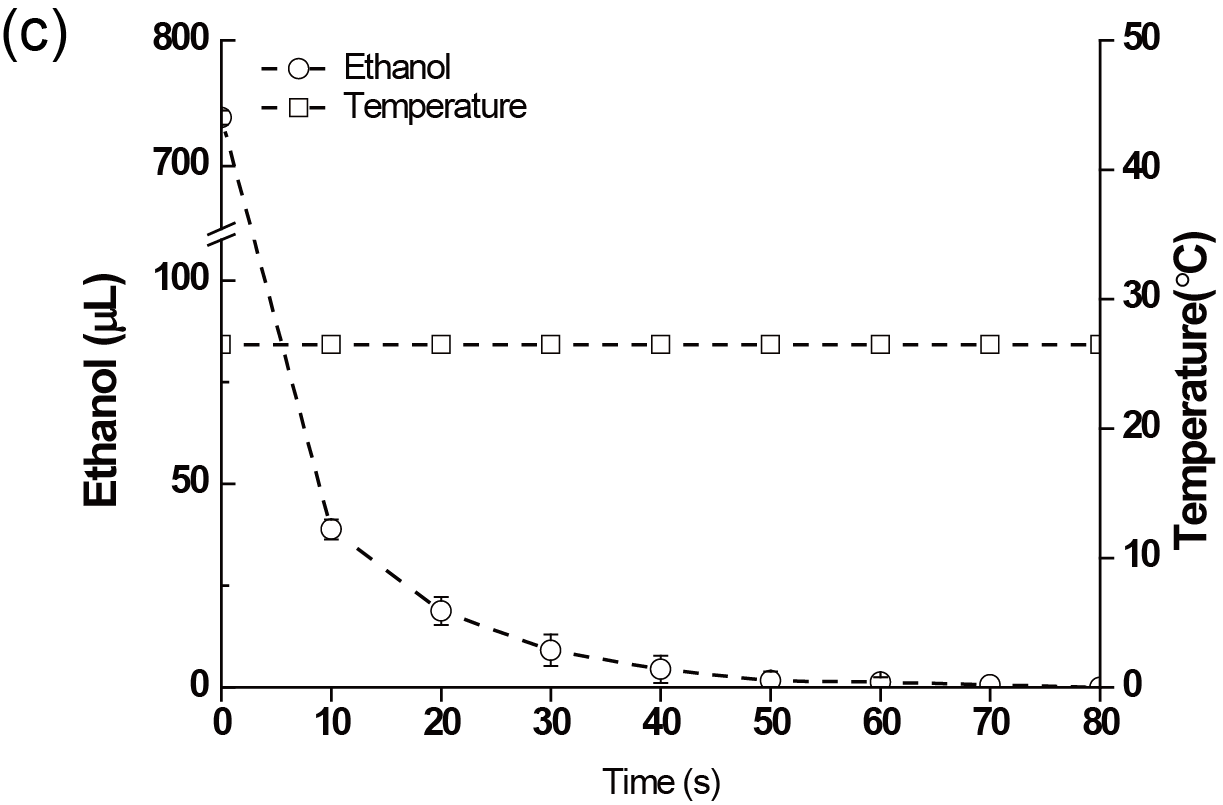

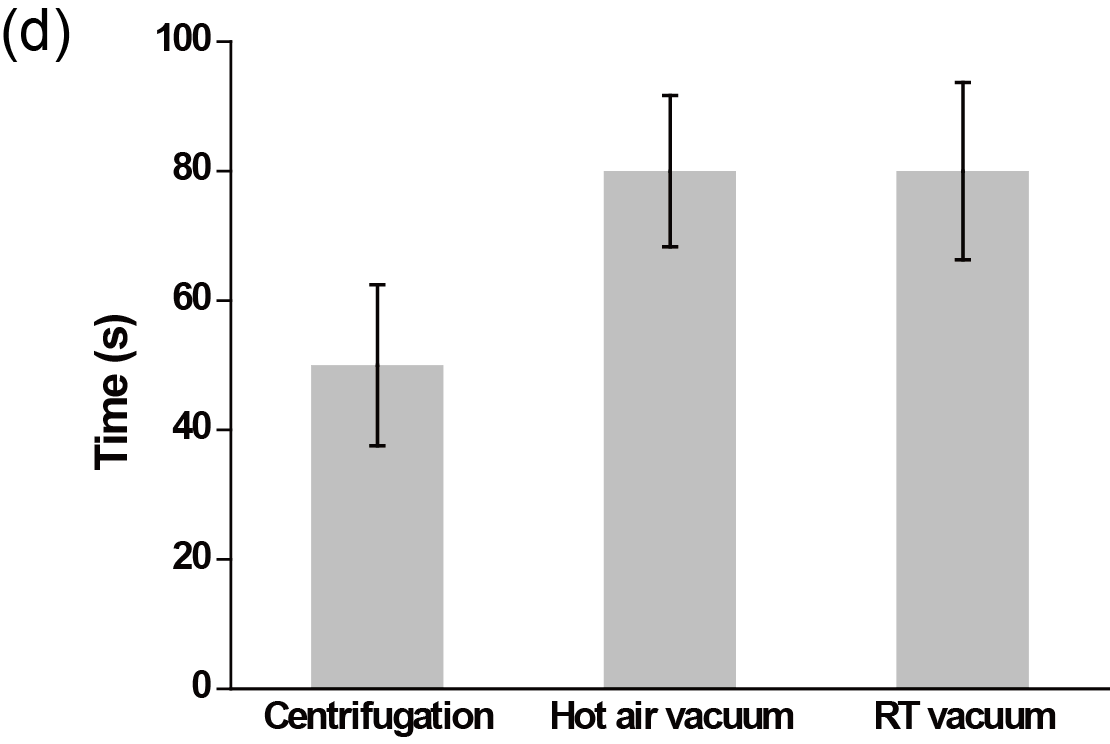


**Supplementary Figure 2. Results of qPCR assay.** Amplification curve and standard curve in qPCR assay with the following reference genes: (a) TERT, (b) GAPDH, (c) NAGK, and (d)RPPH1.


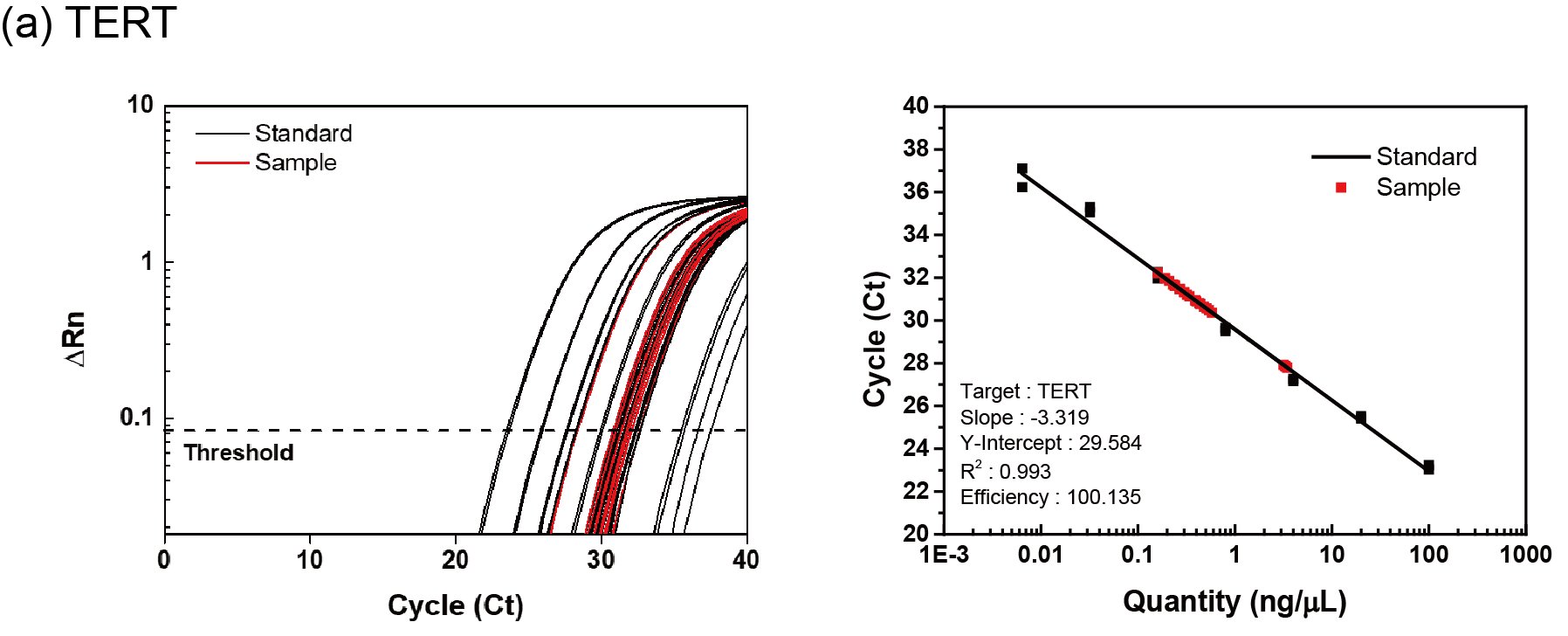


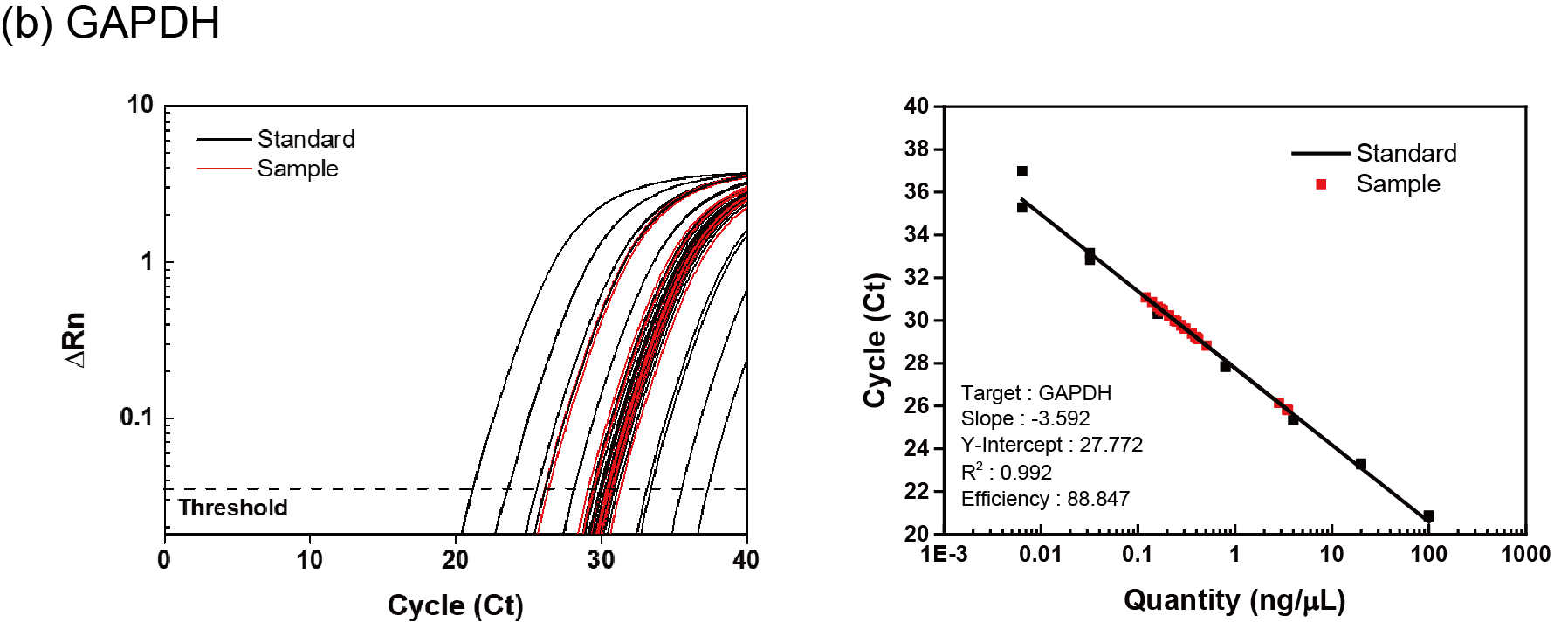


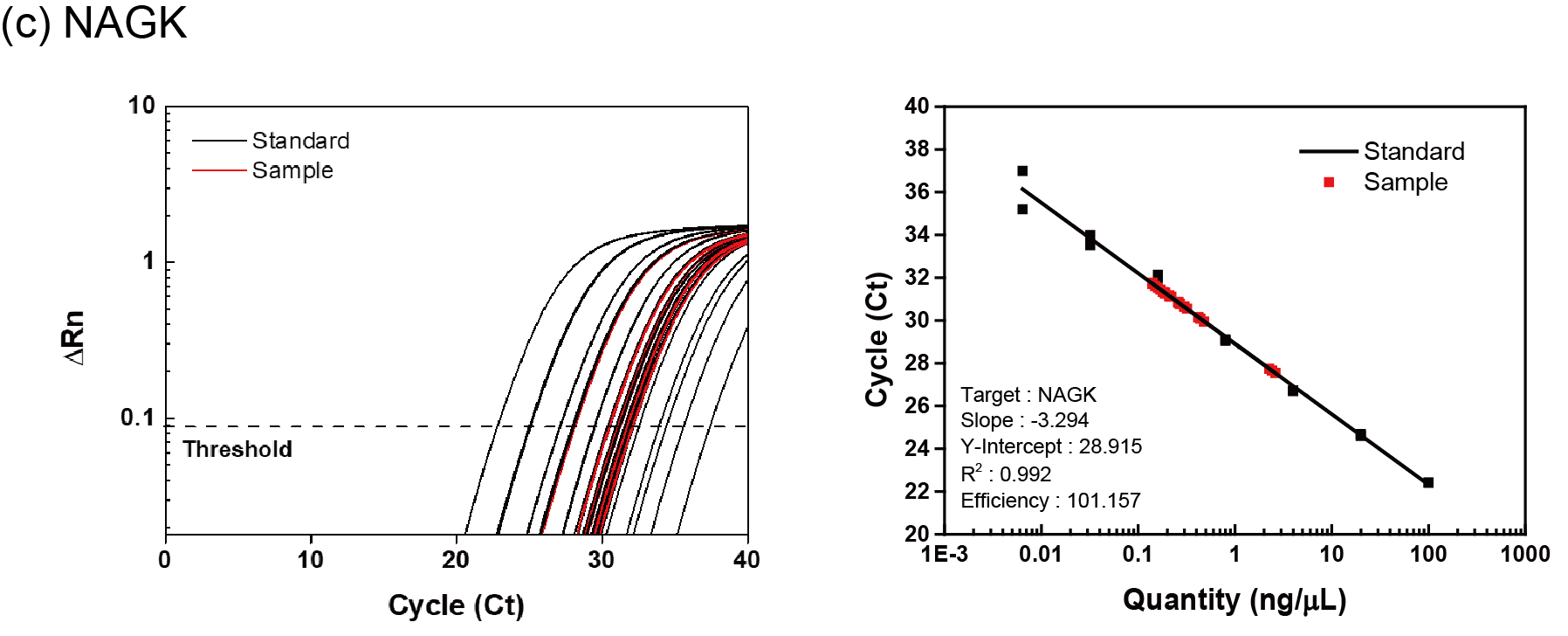


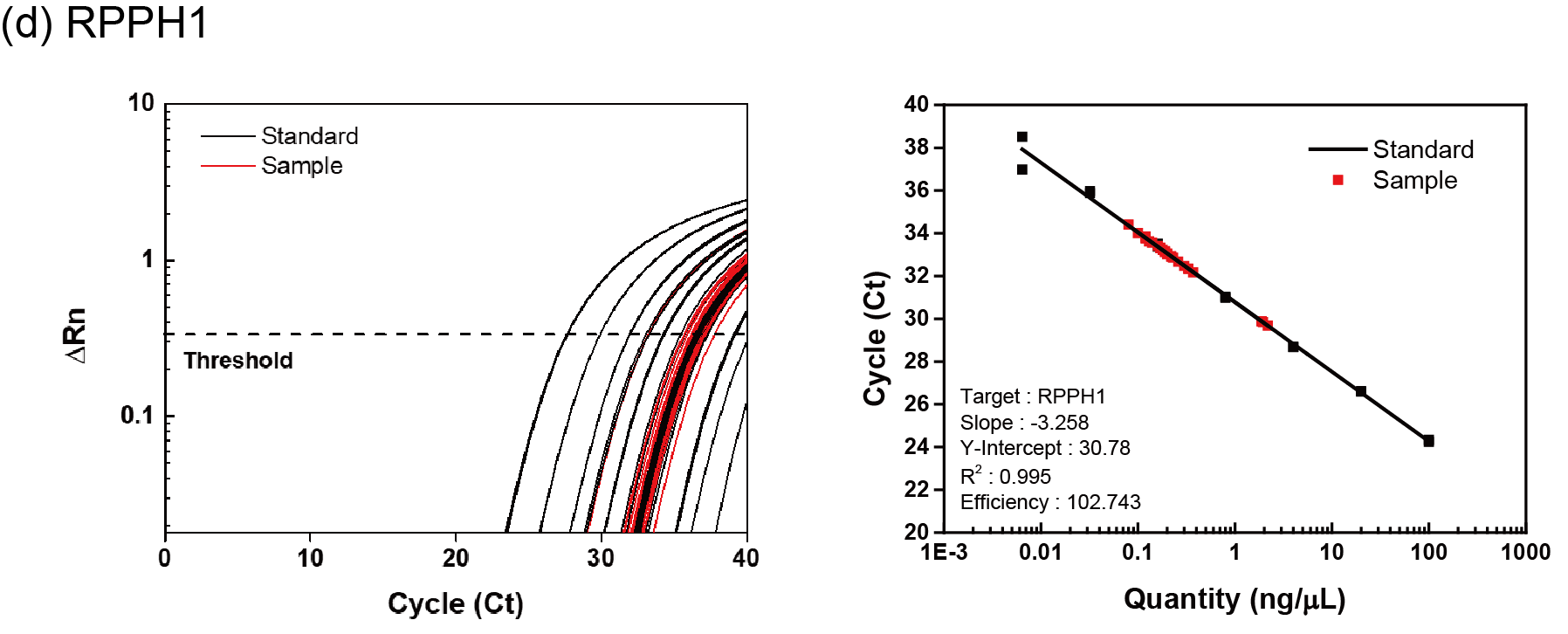


**Supplementary Figure 3**. Size analyses of extracted DNAs using a electrophoretic device (Bioanalyzer 2100). Fig. 3(a) and 3(b) indicate results of QIAamp and PIBEX, respectively. Clear three peaks indicate lower maker (35 bp), upper marker (10,380 bp), and cfDNA peak (~170 bp) respectively.


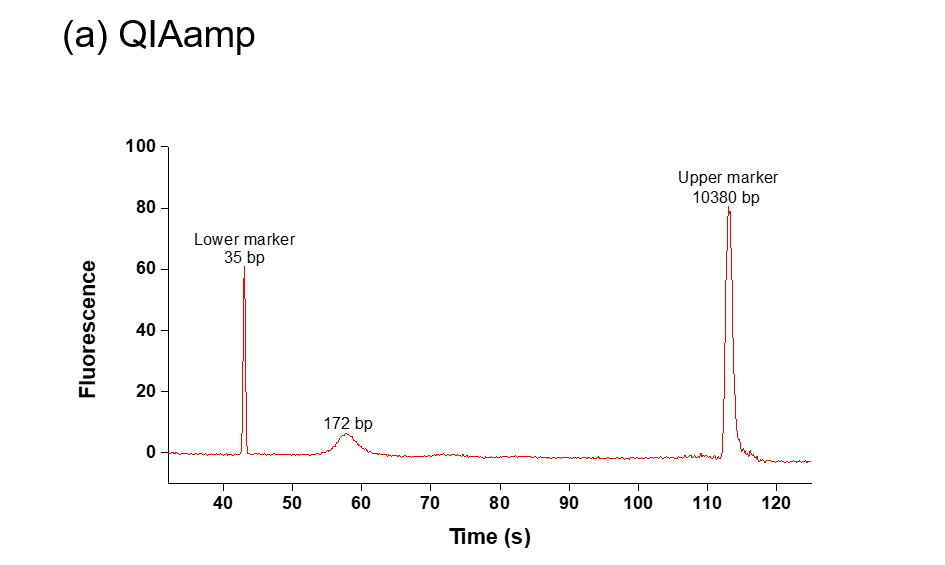


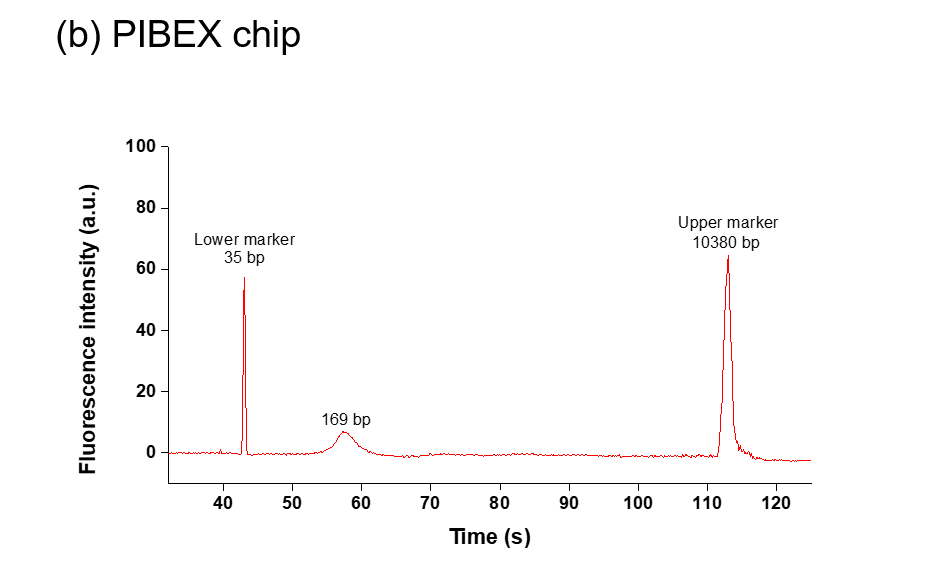


**Supplementary Table 1. Characteristics of study subjects**

| **Characteristic** | **Value** |
| --- | --- |
| **Age - years** | |
| Average | 27.5 |
| Range | 21–35 |
| **Gender** | |
| Male | 6 |
| Female | 1 |
| **Blood type** | |
| A+ | 2 |
| B+ | 2 |
| O+ | 2 |
| AB+ | 1 |

**Supplementary Table 2. qPCR assay information**

| **Gene Symbol / Assay name** | **Genome loci** | **Location** | **Primer/Probe sequence** | **Primer / probe (μM)** | **Probe fluorophore / quencher** | **Efficiency** | **R^2^** |
| --- | --- | --- | --- | --- | --- | --- | --- |
| TERT | 5p15.33 | NC_000005.9  (1253282..  1295178,  complement) | F: CCTCACATAAATGCTACCAAAC | 0.9 | FAM / BHQ-1 | 100.135 | 0.993 |
|  |  |  | R: TTCCAAGAAGGAGGCCATAGTC | 0.9 |  |  |  |
|  |  |  | P: AAGAAATGAACAGACCCATCCCCCAGG | 0.25 |  |  |  |
| RPPH1 | 14q11.2 | NC_000014.8  (20811230..  20811570,  complement) | F: GCGGAGGGAAGCTCATCAG | 0.9 | FAM / BHQ-1 | 102.743 | 0.995 |
|  |  |  | R: GGACATGGGAGTGGAGTGACA | 0.9 |  |  |  |
|  |  |  | P: CACGAGCTGAGTGCG | 0.2 |  |  |  |
| GAPDH | 12p13 | NC_000012.11  (6643585..  6647537) | F: AGGTTTACATGTTCCAATATGATTCCA | 0.45 | FAM / BHQ-1 | 88.847 | 0.992 |
|  |  |  | R: ATGGGATTTCCATTGATGACAAG | 0.45 |  |  |  |
|  |  |  | P: CCGTTCTCAGCCTTGACGGTGC | 0.225 |  |  |  |
| NAGK | 2p13.3 | NC_000002.11  (71295408..  71305998) | F : TGGGCAGACACATCGTAGCA | 0.2 | FAM / BHQ-1 | 101.157 | 0.992 |
|  |  |  | R : CACCTTCACTCCCACCTCAAC | 0.2 |  |  |  |
|  |  |  | P : TGTTGCCCGAGATTGACCCGGT | 0.1 |  |  |  |
